# Knowledge of Metabolic Syndrome Among Third and Sixth Year Medical Students in Saudi Arabia

**DOI:** 10.7759/cureus.73951

**Published:** 2024-11-18

**Authors:** Sami Al-Nasser, Aamir Omair, Mishari Alqahtani, Ziyad Aljabr, Abdulrahman Alshehri, Abdulaziz Alohali, Mohammed Alsalem, Nazish Masoud, Moeber Mahzari

**Affiliations:** 1 College of Medicine, King Saud bin Abdulaziz University for Health Sciences, Riyadh, SAU; 2 College of Medicine, King Abdullah International Medical Research Center, Riyadh, SAU; 3 Health Affairs, Ministry of the National Guard, Riyadh, SAU; 4 Department of Biostatistics, Georgia Southern University, Statesboro, GEO

**Keywords:** education, knowledge, medicine, metabolic syndrome, saudi arabia, students

## Abstract

Background: Metabolic syndrome has become a public health challenge, and its prevalence is increasing among young adults. Little is known about the knowledge of medical students about this syndrome. The study aimed to assess the level of knowledge of the medical students about conditions relevant to metabolic syndrome.

Methods: A cross-sectional survey was administered using a self-administered questionnaire to students in a medical college in Saudi Arabia. The survey was divided into four sections: diabetes, adiposity, hypertension, and hypercholesterolemia. Students’ responses were gathered and interpreted as adequate knowledge (81-100%), moderate knowledge (51-80%), and low knowledge (≤50%). Independent samples t-test and Chi-square tests were done to examine the association between students’ knowledge with the academic year (third and sixth years). The level of significance was set at 0.05.

Results: There were a total of 224 respondents, 102 (46%) from the third year and 122 (54%) from the sixth year. More than 70% of students correctly identified symptoms, complications, and risk factors of diabetes, adiposity, and hypertension, and 90% were aware of different types of diabetes. There were some false beliefs held by students such as 91% of third-year students believed that fatigue is a frequent symptom of high serum cholesterol compared to 70% of sixth-year students (p <0.001). Likewise, 36% of students falsely identified liposuction as the best possible treatment in adiposity therapy. The sixth year of study and family history of hypercholesterolemia was found to be positively associated with a higher knowledge score for most categories.

Conclusion: The study concludes that the student's knowledge about conditions relevant to metabolic syndrome can be enhanced and preventative measures can be implemented to raise awareness about the syndrome.

## Introduction

Metabolic syndrome is a collection of interrelated physiological, clinical, and metabolic factors, including central obesity, hypertension, elevated plasma glucose, and dyslipidemia [1-4], that are associated with a high risk of developing cardiovascular diseases, type 2 diabetes mellitus, cerebrovascular diseases, and all-cause mortality [5]. Individuals with metabolic syndrome are at a twofold higher risk of developing cardiovascular diseases over 5-10 years than individuals without [6]. Historically, the first international organization to introduce a comprehensive definition of metabolic syndrome was the World Health Organization (WHO) in 1998 [7]. The WHO definition mandates the presence of insulin resistance, impaired glucose tolerance, or type 2 diabetes mellitus, along with at least two of the following parameters: elevated blood pressure, elevated triglycerides, low levels of high-density lipoprotein (HDL) cholesterol, obesity (based on waist\hip ratio or body mass index), and microalbuminuria [7]. In 2001, the National Cholesterol Education Program’s Adult Treatment Panel III (ATP-III) published new modified criteria, in which the presence of at least three of the following factors, if present simultaneously, is diagnostic for metabolic syndrome: increased waist circumference, elevated triglycerides, reduced HDL cholesterol, high blood pressure, and elevated fasting glucose [8]. In 2005, the International Diabetes Federation (IDF) introduced a new definition with the same factors as the ATP-III criteria, but the existence of central obesity (increased waist circumference) is mandatory in the IDF criteria [9].

Metabolic syndrome has become a public health and clinical challenge worldwide following urbanization, unhealthy diets, sedentary lifestyles, and increasing obesity [6]. The underlying etiology of this syndrome has been linked to central obesity and insulin resistance [10]. Current literature indicates that metabolic syndrome is prevalent and increasing over time [11]. According to the IDF, approximately 25% of the world’s population has metabolic syndrome, and some recent studies have shown that its prevalence ranges from 10% to 84% depending on the criteria used, age, ethnicity, level of urbanization, and gender of the studied population [12]. The National Health and Nutrition Examination Survey suggests that 34.5% and 39.0% of US adults aged 20 years and over have metabolic syndrome based on the ATP-III criteria and IDF criteria, respectively. In another study targeting the Irish population, the prevalence of metabolic syndrome was 13.2% using the ATP-III criteria and 21.4% using the IDF criteria [13]. Countries in the Middle East and North Africa (MENA) region have reported a high prevalence of metabolic syndrome. In Tunisia, the prevalence of metabolic syndrome has been reported to be 24.3% and 45.5% based on the ATP-III criteria and IDF criteria, respectively [14]. In Gulf countries, metabolic syndrome prevalence ranges from 21.0% in Oman to 39.6% in the United Arab Emirates based on the ATP-III criteria [15,16]. According to a study conducted locally in 2018, the prevalence of metabolic syndrome in Saudi Arabia was 39.9% based on ATP-III criteria and 31.6% based on IDF criteria, which makes Saudi Arabia one of the leading MENA countries in terms of metabolic syndrome prevalence [17]. The study findings were consistent with the high prevalence of diabetes, and obesity that affects approximately one-third of Saudi Arabia’s population, as diabetes and dyslipidemia collectively are a part of metabolic syndrome [18].

Prevention and early risk-factor detection are crucial to prevent or reduce the development of metabolic syndrome, consequently preventing its complications, such as cardiovascular complications later in life [19]. Furthermore, college students should be aware of conditions related to metabolic syndrome so that preventive measures and lifestyle modifications can be implemented at an early age. However, a lack of awareness and knowledge at an early age might lead to a high risk of being undiagnosed until cardiovascular and diabetes complications occur. Medical students, being the custodians of the nation’s future health, must be aware of newly developing health concepts including metabolic syndrome. At present, no local study has evaluated medical students’ knowledge of metabolic syndrome in Riyadh, Kingdom of Saudi Arabia (KSA). As KSA has a high prevalence of metabolic syndrome, these students must have a deep understanding of the condition, which must be first assessed.

The objective of this study was to compare medical students’ knowledge of metabolic syndrome based on their year of study (third and sixth) and stream (stream 1 and stream 2) in Riyadh, KSA. Stream 1 students comprised students who enrolled in medical school directly after graduating from high school, whereas stream 2 students enrolled in medical school after completing a bachelor’s degree in a science or medical-related subject. The study also aimed to assess the knowledge of students with family members who have diabetes, hypertension, and hyperlipidemia compared to students with family members who do not have diabetes, hypertension, or hyperlipidemia. The findings of this study may have implications for preventative measures and health education of students to improve their health and that of the community.

## Materials and methods

Study design, area, and setting

A cross-sectional study was conducted at King Saud bin Abdul-Aziz University for Health Sciences (KSAU-HS) in Riyadh, Saudi Arabia, after receiving approval from the Institutional Review Board (IRB). The IRB, King Abdullah International Medical Research Center (KAIMRC), Riyadh issued approval SP21R/303/06. Unlike other parts of the world, the higher education system in Saudi Arabia has segregated colleges for male and female students. The faculty is primarily male in male colleges and female in female colleges. Students were given instructions about the purpose of the questionnaire used to collect data in this study. The study population included 350 medical students in total, and from 350 there were 180 in the sixth year and 170 in the third year. The Inclusion criteria included all medical students who are in their third or sixth year at KSAU-HS in Riyadh. There were no exclusion criteria. The participants were chosen by non-random convenient sampling technique including all male medical students in the third and sixth year at KSAU-HS, Riyadh. Students who agreed to participate were asked to fill out a consent form. The participants were chosen by non-random convenient sampling based on who was available and willing to participate on the day of data collection. The data collection was conducted during on-campus, problem-based learning sessions conducted weekly for the students of the college.

Data collection process

A paper-based questionnaire was taken from a validated study and modified based on the objectives and relevance to our study [20]. The modification included reducing the item count from 90 to 43 items. Content validity and face validity were assessed by senior research members who were not part of the original research. Comments on changing the wording of a few questions were considered. The modified questionnaire was tested in a pilot study of 50 students. The sections in the modified questionnaire were the same as those of the original questionnaire. There were four sections: diabetes, adiposity, hypertension, and hypercholesterolemia. Reliability testing showed that Cronbach’s alpha of the pilot study was 0.83. The final questionnaire was later used for data collection for the current study.

The final questionnaire included 43 items covering students’ knowledge about conditions relevant to metabolic syndrome, as well as the effects and treatment options for these conditions. The sections on diabetes, adiposity, hypertension, and hypercholesterolemia included 17, 8, 12, and 6 items, respectively. The possible response options to the questions were “true,” “false,” and “do not know.” Correct answers were given one point each, whereas incorrect and “do not know” answers were given zero points. The total score for the questionnaire ranged from 0 to 43 depending on how many points were identified correctly. Students’ knowledge of each question was categorized as good if 81%-100% of students answered the question correctly; fair if 51%-80% of the students answered the question correctly; and low if ≤50% of the students answered the question correctly. All the questionnaires were assigned a serial number to ensure the confidentiality of the participants.

Data analysis

Analysis was performed with IBM SPSS Statistics for Windows, version 22 (IBM Corp., Armonk, NY). Frequencies and percentages were generated for categorical variables such as batch, family history of diabetes mellitus, hypertension, and hypercholesterolemia, whereas mean and standard deviation were calculated for age. Descriptive analyses were performed for all students who were chosen for the study. Independent samples t-tests were done to examine associations between students’ responses and scores for each category of the questionnaire, as well as to assess associations between the demographic data and the scores of the four sections of the questionnaire. The Chi-squared test was used to examine whether two categorical variables are independent in influencing the test statistic (academic year in college, presence of conditions relevant to metabolic syndrome). For all tests, a p-value of <0.05 was considered to indicate a statistically significant association.

## Results

A total of 224 respondents completed the survey, including 102 (46%) third-year and 122 (54%) sixth-year students. The majority of the students, i.e., 205 (92%) were from stream 1 (i.e., high school graduates at entry into medical college), whereas 19 (8%) were from stream 2 (i.e., bachelor’s degree in other health sciences before entry into medical college). The mean age of the students was 22.4 ± 2.0 years (Table 1). There were 135 (60%) students who reported chronic disease as being present in at least one of their immediate family members, as compared to hypertension reported by 94 (42%), diabetes reported by 86 (38%), and hypercholesterolemia reported by 64 (29%).

**Table 1:** Baseline characteristics of the respondents (N=224). Stream 1: High-school graduates; Stream 2: Bachelor’s degree from other health sciences; SD: standard deviation; N: number of participants; %: percentage.

Study variables		N	%
Age (Years)	Mean ± SD	22.4 ± 2.0	
Academic year	Third year	102	46%
	Sixth year	122	54%
Stream	Stream 1	205	92%
	Stream 2	19	8%
Any chronic disease present in the family:		135	60%
Diabetes		86	38%
Hypertension		94	42%
Hypercholesterolemia		64	29%

Based on the 43 items of the questionnaire, the knowledge score regarding metabolic syndrome was totaled after giving a score of one for each correct answer. The scores were also totaled for each of the sub-sections related to diabetes (seventeen questions), adiposity (eight questions), hypertension (twelve questions), and cholesterol (six questions). The total score and the scores for each sub-section were compared in terms of academic year, stream, and having any chronic disease in the family, as shown in Table 2. There was a significant difference in the knowledge scores by academic year. The sixth-year (i.e., final year) students had higher total scores (28.1 ± 7.5) compared to the third-year students (26.0±7.5) (p=0.04). The sixth-year students also had higher scores for diabetes (p=0.01), adiposity (p=0.01), and cholesterol (p=0.04) sub-sections, but no difference was observed for the hypertension sub-section (p=0.82). A significant difference was observed between the high-school entry (stream 1) and graduate-level entry (stream 2) groups only for the cholesterol sub-section, with the latter having a higher score (3.8 + 1.0) than the former (3.1+1.4) (p=0.04). Comparing the scores between stream 1 and stream 2 students across the other categories revealed only a borderline difference for total score (p=0.06) and those of the adiposity (p=0.06) and hypertension (p=0.07) sub-sections. No significant difference (p>0.05) was observed in the knowledge scores for all categories when comparing those students with or without any chronic disease in the family (Table 2).

**Table 2:** Comparison of knowledge scores for different categories by academic year, stream, and presence of any chronic disease in the immediate family. Stream 1: High-school graduates; Stream 2: Bachelor’s degree from other health sciences; p-values determined by independent samples t-test; * significant at p<0.05; SD: standard deviation; n: number of participants; %: percentage.

Knowledge score		Academic year		Stream		Any chronic disease in the family	
		Third year (n=102) (46%)	Sixth year (n=122) (54%)	Stream 1 (n=205) (92%)	Stream 2 (n=19) (8%)	Yes (n=135) (60%)	No (n=89) (40%)
All questions (Total = 43)	mean ± SD	26.0 ± 7.5	28.1 ± 7.5	26.8 ± 7.6	30.2 ± 6.2	27.3 ± 7.4	26.8 ± 7.8
	p-value	p = 0.04*		p = 0.06		p = 0.66	
	t-statistic	-2.052		-1.885		-0.433	
Diabetes (Total = 17)	mean ± SD	10.7 ± 2.8	11.8 ± 3.2	11.2 ± 3.1	11.9 ± 2.5	11.3 ± 3.1	11.2 ± 3.1
	p-value	p = 0.01*		p = 0.37		p = 0.88	
	t-statistic	-2.653		0.898		-0.025	
Adiposity (Total = 8)	mean ± SD	4.3 ± 2.0	5.1 ± 2.0	4.7 ± 2.0	5.6 ± 2.1	4.9 ± 1.9	4.6 ± 2.3
	p-value	p = 0.01*		p = 0.06		p = 0.30	
	t-statistic	-2.580		-1.903		-0.065	
Hypertension (Total = 12)	mean ± SD	8.0 ± 2.9	7.9 ± 2.9	7.8 ± 2.9	8.9 ± 2.4	8 ± 2.8	7.8 ± 3.0
	p-value	p = 0.82		p = 0.07		p = 0.76	
	t-statistic	0.234		-1.926		-0.317	
Cholesterol (Total = 6)	mean ± SD	3.0 ± 1.4	3.3 ± 1.3	3.1 ± 1.4	3.8 ± 1.0	3.2 ± 1.4	3.2 ± 1.4
	p-value	p = 0.04*		p = 0.04*		p = 0.85	
	t-statistic	-2.083		-2.095		-1.582	

Table 3 compares the correct response rates for the 17 items related to diabetes knowledge by academic year. Nine of the 17 items were answered correctly by more than 156 (70%) of the total 224 students, whereas there were four items that were answered correctly by less than 112 (50%) of the students. A greater proportion of sixth-year students had correct responses for six of the items related to diabetes compared to third-year students (the correct response for all six was “False”); these included “too much sugar enters the cells” (p=0.005), “with diabetes, sugar cannot move in the blood” (p=0.02), “individuals with diabetes must have insulin shots” (p=0.048), “increased alertness is a frequent symptom of diabetes” (p<0.001), “diabetic individuals may only eat special kinds of sweets” (p=0.001), and “poor appetite is a frequent symptom of diabetes” (p=0.009). For the item, “there are several different types of diabetes” (correct response=”True”), a greater proportion of third-year students (98%) answered correctly compared to the sixth-year students (87%) (p=0.002).

**Table 3:** Correct answers by academic year for knowledge questions on diabetes. p-values determined by Chi-squared test; * significant at p<0.05; n: number of participants; %: percentage.

Knowledge questions on diabetes	Correct option: (T) = True/(F) = False	Total (n=224)		Academic year				Chi-square value	p-value
				Third year (n=102)		Sixth year (n=122)			
		n	%	n	%	n	%		
1. There are several different types of diabetes	(T)	206	90%	100	98%	106	87%	9.352	0.002*
2. Eye disorders can be consequences of diabetes	(T)	201	88%	90	88%	111	91%	0.455	0.5
3. Hereditary factors play a major role in the development of diabetes	(T)	196	86%	93	91%	103	84%	2.314	0.13
4. Frequent urination is a classic symptom of diabetes	(T)	194	85%	87	85%	107	88%	0.278	0.6
5. Pregnant women have an increased risk of acquiring diabetes.	(T)	177	78%	80	78%	97	80%	0.039	0.84
6. Arteriosclerosis is one of the sequelae of diabetes	(T)	171	75%	76	75%	95	78%	0.347	0.56
7. Hereditary factors play only a minor role in the development of diabetes	(F)	165	74%	72	71%	93	76%	0.911	0.34
8. Pregnant women have a reduced risk of acquiring diabetes	(F)	164	73%	75	74%	89	73%	0.009	0.92
9. Diabetes is a predisposing factor for a myocardial infarction	(T)	161	71%	77	75%	84	69%	1.211	0.27
10. With diabetes, too much sugar enters the cells	(F)	141	63%	54	53%	87	71%	8.038	0.005*
11. With diabetes, sugar cannot move in the blood	(F)	139	62%	55	54%	84	69%	5.260	0.02*
12. With diabetes, sugar cannot enter the cells sufficiently	(T)	133	58%	56	55%	77	63%	1.553	0.21
13. Individuals with diabetes must have insulin shots	(F)	115	51%	45	44%	70	57%	3.910	0.048*
14. For some individuals with diabetes, it is not advisable to take insulin	(T)	111	49%	55	54%	56	46%	1.429	0.23
15. An increased alertness is a frequent symptom of diabetes	(F)	99	44%	28	27%	71	58%	21.293	<0.001*
16. Individuals with diabetes may only eat special kinds of sweets for diabetes	(F)	87	38%	27	26%	60	49%	12.061	0.001*
17. Poor appetite is a frequent symptom of diabetes	(F)	68	30%	22	22%	46	38%	6.842	0.009*

Table 4 shows the responses to the eight items related to adiposity. There were two items that were answered correctly by more than 70% of the total 224 students, and three items that were answered correctly by less than 50% of the students. Two items were answered correctly by a greater proportion of sixth-year students compared to third-year students (the correct response for both items was “False”). The two items were, “the terms overweight and adiposity are synonyms” (p<0.001), and “liposuction is the best possible treatment” (p<0.001).

**Table 4:** Correct answers by academic year for knowledge questions on adiposity. p-values determined by Chi-squared test; * significant at p<0.05; n: number of participants; %: percentage.

Knowledge questions on adiposity	Correct option: (T) = True/(F) = False	Total (n=224)		Academic year				Chi-square value	p-value
				Third year (n=102)		Sixth year (n=122)			
		n	%	n	%	n	%		
1. Adipose individuals have an elevated risk of suffering myocardial infarction	(T)	193	85%	89	87%	104	85%	0.188	0.66
2. Adipose individuals are more likely to suffer from arteriosclerosis	(T)	182	80%	82	80%	100	82%	0.090	0.76
3. Adipose individuals have the same risk than non-adipose individuals of suffering a stroke	(F)	152	68%	66	65%	86	70%	0.853	0.36
4. Cessation of breathing while sleeping is a possible consequence of adiposity	(T)	149	65%	71	70%	78	64%	0.803	0.37
5. An excessively, fatty, high-caloric diet is the only factor that determines adiposity	(F)	114	52%	46	45%	68	56%	2.516	0.11
6. The terms “overweight” and “adiposity” are synonyms	(F)	95	43%	29	28%	66	54%	14.985	<0.001*
7. Adiposity can be treated surgically	(T)	94	41%	44	43%	50	41%	0.016	0.74
8. Liposuction is the best possible treatment in adiposity therapy	(F)	81	37%	16	16%	65	53%	34.008	<0.001*

Table 5 shows the responses to the twelve items related to hypertension. Five items were answered correctly by more than 70% of the students, and two items were answered correctly by less than 50% of the students. Two questions were answered correctly by a higher proportion of sixth-year students compared to third-year students (the correct response to both items was “False”); these included, “pregnant women are less likely to suffer from hypertension” (p=0.045), and “pregnant women are as likely to suffer from hypertension compared to non-pregnant women” (p=0.03). A greater proportion of third-year students (88%) responded correctly (True) to “hypertension can cause renal damage” compared to the sixth-year students (75%) (p=0.01).

**Table 5:** Correct answers by academic year for knowledge questions on hypertension. p-values determined by Chi-squared test; * significant at p<0.05; n: number of participants; %: percentage.

Knowledge questions on hypertension	Correct option: (T) = True/(F) = False	Total (n=224)		Academic year				Chi-square value	p-value
				Third year (n=102)		Sixth year (n=122)			
		n	%	n	%	n	%		
1. Hypertension can lead to eye disorders	(T)	185	81%	88	86%	97	80%	1.769	0.18
2. Hypertension can cause renal damage	(T)	181	79%	90	88%	91	75%	6.668	0.01*
3. Individuals with hypertension are less likely to suffer from arteriosclerosis	(F)	168	75%	74	73%	94	77%	0.600	0.44
4. Hypertension is associated with heredity	(T)	166	73%	80	78%	86	70%	1.825	0.18
5. Hypertension can cause dizziness	(T)	164	72%	81	79%	83	68%	3.668	0.06
6. Hypertension can be caused by disorders of the thyroid gland	(T)	150	66%	73	72%	77	63%	1.795	0.18
7. After medication has lowered hypertension, the medication can usually be discontinued	(F)	146	66%	62	61%	84	69%	1.593	0.21
8. Pregnant women are less likely to suffer from hypertension	(F)	141	64%	57	56%	84	69%	4.007	0.045*
9. People with hypertension are as likely to suffer from arteriosclerosis as those with normal blood pressure	(F)	132	60%	56	55%	76	62%	1.255	0.26
10. Pregnant women are as likely to suffer from hypertension as non-pregnant women	(F)	125	57%	49	48%	76	62%	4.578	0.03*
11. For the most part, a concrete single reason of why a patient suffers from hypertension can be determined	(F)	108	49%	47	46%	61	50%	0.342	0.56
12. Hypertension can be caused by cerebral tumors	(T)	107	47%	54%	53%	53%	43%	2.009	0.16

Regarding the six questions related to hypercholesterolemia (Table 6), the item, “fatigue is a frequent symptom of high serum cholesterol” (correct response = False) was answered correctly by 30% of the sixth-year students, as compared to 9% of the third-year students (p<0.001).

**Table 6:** Correct answers by academic year for knowledge questions on hypercholesterolemia. p-values determined by Chi-squared test; * significant at p<0.05; n: number of participants; %: percentage.

Knowledge questions on hypercholesterolemia	Correct option: (T) = True/(F) = False	Total (n=224)		Academic year				Chi-square value	p-value
				Third year (n=102)		Final year (n=122)			
		n	%	n	%	n	%		
1. High serum cholesterol can be treated with medication	(T)	177	78%	79	77%	98	80%	0.277	0.6
2. High serum cholesterol promotes arteriosclerosis	(T)	176	77%	76	75%	100	82%	1.835	0.18
3. High serum cholesterol is not associated with hereditary factors	(F)	147	66%	66	65%	81	66%	0.070	0.79
4. A low-cholesterol diet can supplement therapy for high serum cholesterol	(T)	129	57%	57	56%	72	59%	0.223	0.64
5. Fatigue is a frequent symptom of high serum cholesterol	(F)	45	20%	9	9%	36	30%	14.806	<0.001*
6. High serum cholesterol does not cause acute ailments	(T)	36	18%	15	15%	21	17%	0.259	0.61

## Discussion

The aim of this study was to evaluate the knowledge of conditions relevant to metabolic syndrome among third-year and sixth-year medical students at KSAU-HS in Riyadh, Saudi Arabia. Regarding the students’ knowledge about diabetes, more than half of the total sample correctly answered that there are several different types of diabetes, eye disorders can be complicated by diabetes, hereditary factors can play a minor role in the development of diabetes, and frequent urination is a classic symptom of diabetes. In contrast, less than half of the total sample answered correctly that increased alertness is a symptom of diabetes, low appetite is a symptom of diabetes, and individuals with diabetes can only eat special kinds of sweets designed for those with diabetes. A statistically significant association was noted between the students’ knowledge and their year of study (third year vs. sixth year). A similar study conducted in Tabuk, Saudi Arabia, reported that there was a statistically significant relationship between students’ knowledge of diabetes characteristics and their educational major [20]. As expected, the sixth-year students had higher knowledge of diabetes than the third-year students, which indicated the former’s deeper understanding of diabetes. However, some misconceptions were held by the students regarding some of the questionnaire items. More than half of the students believed that individuals with diabetes can eat special kinds of sweets designed for those with diabetes, which may reflect a lack of knowledge about diabetes management and treatment. Furthermore, a US study conducted at Central Michigan University reported that approximately half of the students falsely believed that diabetic individuals can only eat certain kinds of sweets [21].

In the present study, findings related to questionnaire items related to hypertension indicated that more than half of the students had a good knowledge of hypertension complications and treatment. In addition, most of the students were aware that hypertension can manifest as dizziness. The above-mentioned US study at Central Michigan University also reported that many students knew that hypertension could manifest as dizziness; however, the study also reported that less than half of the students answered correctly about whether pregnant women are less likely to suffer from hypertension compared to non-pregnant women [21]. In contrast, our findings found that more than half of the students answered these questions related to hypertension and pregnancy correctly. This difference may indicate the use of different curriculums between Central Michigan University in the US and KSAU-HS in Riyadh, Saudi Arabia [21]. However, results from the above-mentioned study conducted at Tabuk University agreed with our findings [20]. In our study, no statistically significant difference was found between the third-year and sixth-year students for the total knowledge score regarding hypertension.

When analyzing the students’ knowledge about adiposity risks and complications, more than half of the students answered the related items correctly. In contrast, more than half of the students falsely believed that the terms “overweight” and “adiposity” were synonyms. These terms are used synonymously in social media and daily life, even though the term overweight refers to gaining weight, and adiposity refers to excess body fat. Furthermore, approximately two-thirds of the students falsely believed that the best possible treatment for adiposity is liposuction, indicating low knowledge about the treatment and management of adiposity, which may also be attributed to misconceptions common in social media and the complexity of adiposity management. The Central Michigan University study in the US had similar findings regarding the misuse of the terms adiposity and overweight interchangeably, as well as the best possible treatment for adiposity [21]. In our study, there was a statistically significant difference between the third-year and sixth-year students regarding knowledge of adiposity, where the latter scored higher than the former, which is expected, as they had more extensive scientific background regarding these topics.

Moreover, more than half of the students answered all questions about hypercholesterolemia treatment, management, and complications correctly. Similarly, the study from Tabuk University reported the same results [20]. However, in our study, more than two-thirds of the students falsely believed that fatigue is a frequent symptom of high serum cholesterol, which suggests a lack of understanding of hypercholesterolemia symptoms. The Central Michigan University study in the US reported the same findings, where less than one-third of the students answered the same question related to fatigue and high serum cholesterol correctly [21]. In our study, the sixth-year students had higher knowledge of hypercholesterolemia compared to the third-year students, and this difference was statistically significant.

Both third-year and sixth-year students with an immediate family member with hypercholesterolemia demonstrated higher correct response rates in questionnaire items from the adiposity and hypercholesteremia sub-sections, which can be explained by their more extensive experience and awareness of hypercholesterolemia complications. When comparing medical students from stream 1 and stream 2 of both academic years, a statistically significant difference in correct response rate was only observed in the hypercholesteremia sub-section. As expected, the stream 2 students showed higher knowledge of hypercholesteremia compared to stream 1 students, which we attributed to the increased age and more extensive scientific exposure in the former.

Study limitations

The main limitation of this study was that it included only male medical students, resulting in a small sample size, which limits the generalizability of our findings.

## Conclusions

Our results showed that a family history of hypercholesterolemia had an impact on medical students’ increased knowledge about adiposity and hypercholesterolemia. It is recommended that medical students should be given information about metabolic syndrome risk factors and complications early in their academic years. This will help to increase the knowledge and awareness for the prevention of this syndrome and its complications among them and their community. Future studies should evaluate the knowledge of conditions relevant to metabolic syndrome among other medical students in the country and identify their level of knowledge.
